# Sometimes needs change minds: Interests and values as determinants of attitudes towards state support for the self-employed during the COVID-19 crisis

**DOI:** 10.1177/09589287221106977

**Published:** 2022-07-20

**Authors:** Giuliano Bonoli, Flavia Fossati, Mia Gandenberger, Carlo Michael Knotz

**Affiliations:** 27213 University of Lausanne, Switzerland; 56627 University of Stavanger, Norway

**Keywords:** self-employed, deservingness, welfare state, COVID-19, pandemic

## Abstract

This contribution investigates public attitudes toward providing financial help to the self-employed, a less well-researched area in the otherwise vibrant literature on welfare state attitudes. We analyse to what extent the self-employed themselves soften their general anti-statist stance in times of need, and how the public thinks about supporting those who usually tend to oppose government interventions. To answer these questions, we study public attitudes towards providing financial aid to the self-employed during the lockdowns adopted in response to the COVID pandemic in Switzerland, using survey data collected in the spring and in the autumn of 2020. The results show that most respondents favour the provision of financial support. In addition, the self-employed are the staunchest supporters of the more generous forms of help, like non-refundable payments. We conclude that, when exposed to significant economic risk, need and interests override ideological preferences for less state intervention.

## Introduction

The self-employed have traditionally had an uneasy relationship with the welfare state. Historically, they have sought to avoid inclusion in income- and risk-redistributive arrangements. When they have been included, this has usually been done on minimalist terms, for example with access to limited and flat-rate subsistence benefits (Baldwin, 1990; Mares, 2003). The result is that today, in most welfare states, the self-employed are underprotected against most traditional social risks such as old age, invalidity and unemployment. This outcome is perfectly in line with their overall ideological orientation characterized by adherence to values such as financial self-reliance and scepticism of state involvement in economic and social affairs. Analyses of public attitudes towards the welfare state have indeed consistently shown that the self-employed tend to oppose more social spending and higher levels of state intervention in the broad field of social protection (for example, Mau, 2003; Svallfors, 2004).

In times of crisis, however, even the self-employed have occasionally turned to the state for help. For example, after the Second World War, the self-employed in much of Continental Europe were confident in their capacity for self-reliance and declined to join the social insurance systems that were being developed. However, by the 1960s, as structural economic changes worsened their economic position, they too asked for government aid (for example, Baldwin, 1990: 248–287 on France and Germany; Ferrera, 1993: 240–257 on Italy). In spite of the policies adopted in response to growing demands in those years, the self-employed remain an underprotected group in most welfare states (Spasova et al., 2017) and tend to be overexposed to the risk of (in-work) poverty (Crettaz, 2013; Halleröd et al., 2015).

The complex relationship between the self-employed and the welfare state raises a range of questions concerning their social policy preferences, but also the reaction of the public when the self-employed appeal to the state for help. In difficult times, do the self-employed set aside their preference for a small government and ask for state help? And is the public in general supportive of helping the self-employed? If so, on what conditions? Furthermore, does the case of economic support to the self-employed reflect the traditional left–right dimension, or does it cut across it? Particularly interesting here is how left-wing voters view the issue of economic aid to the self-employed: do they keep favouring government intervention even when it concerns those who normally stand on the other side of the political spectrum? And if yes, what type of aid do they prefer?

So far, these are still very much open questions. Despite the large amount of micro-level research on attitudes toward various social protection programmes or the welfare state in general (for example, Blekesaune, 2007; Iversen and Soskice, 2001; Margalit, 2013; Rehm, 2016; Van Oorschot et al., 2017), not much is known about the attitudes of the general public toward social protection *specifically for the self-employed*. The literature on employers’ welfare state attitudes (for example, Mares, 2003; Paster, 2013) has likewise been mostly concerned with their attitudes toward policies for workers or citizens in general, although some studies have looked more in detail at the risk perceptions and policy attitudes of the self-employed (Baldwin, 1990; Ferrera, 1993; Jansen, 2016a). Understanding public attitudes toward policies for the self-employed is important in and of itself, but it will also become increasingly important given the recent (and likely continuing) rise in self-employment associated with the ‘gig economy’ (Abraham et al., 2019).

Therefore, we contribute to closing this gap by studying political attitudes toward financial aid for the self-employed during the COVID-19 pandemic in Switzerland. Here, as in other countries around the world, many self-employed workers were forced to stop their economic activities (for example, running restaurants) because of sudden restrictions of various economic activities. Others saw their levels of activity decline almost completely (for example, travel agents, taxi drivers). In such a context, the lack of social protection for the self-employed became a major policy problem, the main issue being whether taxpayer money should be used to support small businesses and if so on what conditions.

This crisis provided a context in which we can study the policy preferences of the general public in relation to supporting the self-employed as well as the position of the self-employed in welfare state politics. We analyse these issues with original survey data collected at two points in time during the pandemic crisis in Switzerland, first during the first lockdown in the spring of 2020 and then again in the late autumn of 2020 during the much more severe second wave. Our survey included items on preferences toward government aid to small companies of different sizes (up to two and up to 50 employees), in which respondents were asked to rank several different policy options such as no help at all, various types of loans, and a non-refundable payment.

With this article, we contribute to several strands of literature. First, we shed light on the general question of the determinants of social policy preferences (for example, Cusack et al., 2006; Häusermann et al., 2015). Second, we contribute to a much less developed strand of literature on the relationship of the self-employed to the welfare state (for example, Baldwin, 1990; Ferrera, 1993; Mares, 2003). Finally, we also add to the recent literature on the impact of the COVID-19 crisis on political attitudes (for example, Sabat et al., 2020; Blumenau et al., 2021).

The article begins with a discussion of the factors that may influence support for providing financial help to the self-employed and allows us to identify a few hypotheses. It then presents the data and the methods we apply. The next section presents the main results, first in relation to the overall ranking of policy options and then with a focus on key potential determinants of policy preferences. Finally, we conclude by highlighting our contribution to the literature on preference formation in social policy and on the position of the self-employed in welfare politics.

## Theory and hypotheses

In order to generate hypotheses, we rely essentially on two strands of literature. First, we consider scholarship on the determinants of social policy preferences. Second, we concentrate on a small number of studies focusing more specifically on the social policy preferences of the self-employed.

### The determinants of social policy preferences

The literature on the determinants of social policy preferences has identified two broad categories of determinants: interest (that is, risk exposure, income) and ideology. Historically, exposure to social risk has been a major determinant of group mobilization in favour of the introduction of social insurance or other redistributive programmes (Baldwin, 1990; Ferrera, 1993). More recently, work on social policy preferences based on public attitude data reached similar conclusions. Risk exposure has been found to be a major determinant of social policy preferences (Cusack et al., 2006; Häusermann et al., 2015; Iversen and Soskice, 2001; Rehm, 2009). This view has been shown to be valid for both objective measurements of risk exposure, subjective perceptions of economic insecurity (Cusack et al., 2006), as well as socioeconomic status (Häusermann et al., 2015; Iversen and Soskice, 2001; Rehm, 2009, 2016).

Regarding ideology, it is well-known that left-wing voters and parties have traditionally mobilized for more extensive and generous forms of social protection (for example, Korpi, 1983; Huber and Stephens, 2001). Furthermore, in the literature on public attitudes towards social policies, the positioning on the left–right axis has been found to be a powerful determinant of attitudes towards the welfare state (for example, Mau, 2003). The recent shift towards a social investment welfare state has not fundamentally altered this tendency (Häusermann, 2012; Huber and Stephens, 2006).

Other studies have focused on the role of social class as a determinant of social policy preferences (see for example, Svallfors, 1995, 2004). The notion of social class combines risk-exposure and ideology, and in a way, this makes sense. While they are different concepts, risk-exposure and ideology need not be orthogonal in the real world, and are in fact likely to covary (see also Wehl, 2019). Social class, as a result, is a powerful determinant of support for redistribution and for most social policies, with manual workers being among the strongest supporters and the self-employed constituting the main opponents (Svallfors, 2004).

Assumptions with regard to the role of risk-exposure and ideology in preference formation on social policy can be used to generate a few simple hypotheses. On the basis of a risk-exposure-based understanding of policy preferences formation, we would expect the self-employed to be more supportive of financial aid for the self-employed than employees. On the basis of ideology, we would expect left-wing respondents to support more financial aid for the self-employed.

As argued above, risk exposure and ideology are unlikely to be orthogonal. Rather, the self-employed can be expected to be among the staunchest opponents of social policies under normal circumstances, a result which is compatible with both risk-exposure and ideology-based hypotheses. However, during the pandemic, the position of the self-employed on the risk-exposure axis changed dramatically. Did this have an impact on their policy preferences? Did a significant change in risk exposure overrule ideology as a determinant of policy preferences in this particular situation? Before trying to answer this question, we will consider the literature on policy preferences of the self-employed as a group, particularly when exposed to economic insecurity.

### The preferences of the self-employed

Traditionally, the self-employed have mobilized for and their interests have been championed by right-wing, anti-statist and, sometimes, populist parties, that is, the parties who in normal circumstances oppose state intervention, redistribution and high levels of social protection. In electoral sociology, small business owners, sometimes referred to as part of the ‘petty bourgeoisie’ are among the most fervent supporters of the political right (for example, Scase and Goffee, 1981). However, in recent years, the composition of the self-employed as a social group has changed (Buschoff and Schmidt, 2009). While historically the self-employed as a group consisted mostly of small business owners with anti-statist values and a strong preference for private initiative and self-reliance, increasingly, this group includes also highly educated socio-cultural professional people who tend to vote and mobilize for post-material issues and left-wing parties (Oesch, 2006). These ‘new self-employed’ workers, active in various fields such as the arts, culture, journalism, translation services, tend to vote in line with similar socioeconomic groups with employee status (Jansen, 2016a). As a result of this socio-structural transformation, the kind of historical association between the self-employed and right-wing parties may be waning. Empirical research suggests that there has been a change in the overall political orientation of the self-employed over the last few decades; however, as a whole, the self-employed remain a group with right-wing political attitudes (Barisone and De Luca, 2018).

The finding of an overall tendency among the self-employed to position themselves to the right is compatible with the results of studies on welfare attitudes (Mau, 2003; Svallfors, 1995, 2004). Low levels of support by the self-employed are visible across the range of social policies, but particularly for unemployment benefit (Mau, 2003: 136). In the literature on welfare deservingness the self-employed are found to be stricter in the application of conditionality in terms of identity, control, attitude and need (Meuleman et al., 2020), suggesting stronger concerns for ‘free riders’ than in the rest of the population. Cusack et al. (2006) show that the self-employed are consistently found among the groups that are least supportive of redistribution. Their argument is that since the self-employed often rely on cheap labour for their operations, they are likely to oppose generous (and costly) social protection schemes (Cusack et al., 2006: 372). Probably for similar reasons, the self-employed are also among the most critical of job security regulations (Emmenegger, 2009), and they favour demanding activation policies (Rossetti et al., 2020). The self-employed (unless they do not have employees) are also less likely to favour the introduction of a basic income than employees (Shin et al., 2020). The self-employed are more likely than any other group defined by work status to believe that the welfare state represents too much of a strain on the economy (Tóth et al., 2020: 171).

While the point that the self-employed hold anti-welfare views is clearly confirmed by several empirical studies, the question of their policy preferences when exposed to economic insecurity has been less researched. However, the available evidence suggests that when exposed to economic insecurity, the self-employed are more likely to support social protection. Jansen (2016b) for example found that the self-employed in general were less likely to support social policies, but if they perceived their income or their employment as insecure, they were more supportive of social policies than permanent employees (Jansen, 2016b: 397). This is true whether they have employees or not.

A recent study on the extension of social protection coverage to currently unprotected groups found that the employment status ‘self-employed’ was most strongly associated with the perception of being inadequately covered against the main social risks (73%), against 42% of full-time employees on open ended contracts (Codagnone et al., 2018: 76). However, when asked about joining a voluntary unemployment protection scheme, the self-employed, together with entrepreneurs, were among the least enthusiastic, but the difference with other employment statuses is small (p. 90). According to the authors, these partly unexpected results may be related to the impact of the Great Recession, which has increased economic insecurity across the board (p. 102). Overall, the evidence discussed in this section suggests that when (anti-statist) values and (pro-welfare) interests collide, the latter is dominant.

### Hypotheses

The COVID-19 crisis created a totally new situation for the self-employed. Economic restrictions meant that the position of the self-employed in relation to risk exposure changed suddenly and dramatically, as did their ability for self-reliance. However, based on theory alone, it is rather difficult to formulate clear hypotheses with regard to how risk exposure and ideology will impact on preferences concerning financial support for the self-employed. As a result of theoretical indeterminacy, we will consider a range of contrasting hypotheses and try to settle these questions empirically.

**H1**: The self-employed will be the strongest supporters of state-provided financial help to the self-employed.

This hypothesis assumes that faced with a sudden increase in risk-exposure, the self-employed abandoned their traditional preference for limited state intervention in the economy and now support using tax payers’ money to keep their businesses alive. Non-self-employed individuals may also support state help for the self-employed out of solidarity, but the support will be stronger among the self-employed because of self-interest.

**H2**: The self-employed will be the weakest supporters of state-provided financial help to the self-employed.

In this case the strong ideological opposition to state intervention among the self-employed will prevent them from supporting the most generous forms of state help, and possibly lead them to prefer loans to non-repayable payments in order to limit the costs to the taxpayers and thus be consistent with their historical preference for small government.

**H3**: Left-wing voters will be more supportive of state-provided financial help for the self-employed.

In this case, we assume that the general preference among left-wing voters for more state support applies also when the self-employed are in need. Solidarity for those in need is an important value among left-wing voters, and this could have played a role during the COVID-19 crisis in relation to the objective difficulties which the self-employed had to face.

**H4**: Right-wing voters are more likely to support help for the self-employed.

Alternatively, we can hypothesize that the historical connection between the self-employed and the right will induce right-wing respondents to be more supportive of state help for the self-employed. After all, the self-employed are the historical allies of the right-wing parties (for example the populist Swiss people’s party, SVP-UDC). Loyalty among these groups could prompt right-wing voters to support more help for the self-employed.

The relationship between left–right positioning could be U-shaped, in which case both H3 and H4 would be confirmed, with voters both on the left and on the right end of the spectrum being more favourable to generous support to the self-employed, but for different reasons.

## The social policy package for the self-employed in Switzerland during the COVID-19 lockdowns

Like in other welfare states, the self-employed in Switzerland are generally less protected than salaried employees. The self-employed are not covered by unemployment insurance, and as a result they were not eligible for the temporary unemployment benefit when the crisis struck. This benefit was largely used by employees during the lockdowns and provided them with a replacement income.

During the lockdowns, financial help for the self-employed came in two different forms. First, temporary unemployment benefit was extended to the self-employed so that they could access income replacement benefits. This was a fairly uncontroversial decision that was adopted very early on in the crisis. However, it quickly became clear that for self-employed workers, an income replacement benefit would not be enough to see them through the crisis. Self-employed workers run businesses and, in many cases, they have extra expenses, such as rent and other fixed costs. To address this, in March 2020 the government introduced a second type of help for the self-employed, in the shape of an easy to access loan scheme. Even at that time, however, many argued in favour of non-refundable payments for small businesses. In November 2020, the scheme to help small businesses was extended to include precisely non-repayable payments (see Table S1 in the supplementary materials for details).

For our analysis of public attitudes toward help to the self-employed we decided to focus on the second type of help, that is, help to small businesses; this was for a number of reasons. First, the income replacement benefit that was introduced immediately after the adoption of the first lockdown was uncontroversial and there was virtually no debate on the legitimacy of this type of help. In contrast, help to small businesses was subject to much debate and controversy. We reasoned that the second type of state help would be better suited to identify putative differences in the willingness to support the self-employed than the first one. Second, the self-employed who were mostly affected by the lockdown were those whose activities implied physical contact with and among clients, like restaurant owners, hairdressers, and providers of other personal services. These activities generally have significant fixed costs (most notably rent), and so it made sense to focus on help to small businesses as opposed to income replacement benefits.

We are aware that our decision raises a more fundamental conceptual issue regarding whether help to small businesses can be considered as part of a social policy package. In our view the answer depends on the type of business targeted, and particularly its legal status. In the case of the self-employed, we would argue that this is the case. This type of help keeps their potential source of income alive. It is a form of preventative social policy that limits the likelihood of the self-employed becoming fully dependent on the welfare state. In a way, help to small businesses for the self-employed is akin to employment protection legislation for employees, that is, a policy that aims at preventing the risk that a person loses his or her source of livelihood, whether a job or a small business.

During the COVID crisis, the relevance of help to small businesses as a tool to limit the social and economic disruption produced by the lockdowns became evident. In this context, providing income replacement benefits alone would have been pointless for all the self-employed people with a business generating fixed costs. Without help to keep their small business alive, many would have gone bankrupt. So, in our view, the theoretical case for considering help to small businesses as part of a social policy package for the self-employed got even stronger during the lockdowns. Note that a similar stance has sometimes been adopted in research on help to the self-employed during the COVID crisis. For example, overviews of social protection for the self-employed during the COVID-19 crisis also cover help to small businesses in various forms, including loans, non-repayable payments and tax and contribution exemptions (for example, Baptista et al., 2021: 57–60; and OECD, 2020).

## Data and methods

To study attitudes toward providing aid to the self-employed, we use original data from a two-round public opinion survey that was conducted in Switzerland in 2020 between 22 April and 4 May (round 1) and between 19 November and 14 December (round 2). Our survey was administered to a sample that was recruited from an online respondent pool operated by a European public opinion research firm. This respondent pool, in turn, is comprised of volunteer participants living in Switzerland, who receive a small compensation for participating in surveys.

Our respondents (round 1, *N* = 1535; round 2, *N* = 1498) were selected to obtain samples that resemble the Swiss population in terms of residency in the two largest linguistic regions (German- and French-speaking), gender, age and educational attainment. The Supplementary materials include figures showing the demographic composition of our two samples and a comparison to official statistics on a number of relevant dimensions. The notion of ‘self-employed’ was not defined in the survey, but the term is widely used in Switzerland to refer to individuals who own their own business, and it is safe to assume that its meaning was clear for our respondents. The proportion of self-employed in our sample (round 1: 11.63%; round 2: 11.74%) is close to the one in the working population as shown in official statistics (12.6% in 2020).^1^ Additional descriptive statistics about our samples can also be accessed via our online data dashboard.

The survey contained two ranking tasks in which our respondents were asked to rank six different types of government aid to small businesses: (1) a non-repayable one-off payment; (2) a one-off payment repayable when business conditions would improve again; (3) an interest-free loan; (4) a low-interest loan; (5) a one-off payment repayable with low interest after 5 years; and (6) no aid. These were the options that were being discussed in the public debate at the time of the first round of the survey.

Respondents were asked to rank these policy options once for very small establishments (up to two employees) and once for slightly larger firms (up to 50 employees). We adopted these two thresholds for a number of reasons. This distinction allows us to capture possible differences between a self-employed person who runs a very small business, either alone or with one or two employees, and a self-employed person who runs a bigger operation. Solo self-employed people, who are often the focus of the social policy literature are included in our first category. The distinction between a solo self-employed person and those with one or two employees may be less watertight than it appears, as the same person may have employees sometimes, but not always. This is what we wanted to indicate with the formulation; ‘up to two employees’ is a very small business. In addition, during the lockdowns, the type of small businesses that suffered most were restaurants, personal services (for example, hairdressers), retail in non-essential goods, that is, sectors in which having employees is also quite common for the smallest businesses. The order in which the different options were shown was randomized. Respondents were neither allowed to rank two alternatives equally nor to submit incomplete rankings.

As mentioned above, the economic hardship experienced by many small- and medium-sized companies in Switzerland after the lockdown and the early aid programmes introduced by the Federal government were a major topic in the Swiss national news throughout the time span covered by the two rounds of our survey. Our respondents should therefore have been able to make informed choices in our ranking tasks.

### Methods

Our dependent variable is by nature a rank-ordering of different policy-options, which we would ideally model with the rank-ordered logit regression model (following Allison and Christakis, 1994). However, this model relies on the assumption that respondents’ preferences remain stable over the entire ranking task – which means, more simply put, that respondents remain diligent and do not rank what they see as less important alternatives increasingly randomly (Allison and Christakis, 1994: 216–218).^2^ It turned out that this was not the case in our survey.^3^ As a result, we decided to reduce our ranking data to a simple binary dummy indicating which option our respondents ranked first (equivalent to treating our ranking task as an individual choice task in which respondents choose one out of six options) and to model respondents’ preferences via the conditional logit model (McFadden, 1974).

Our core predictors are the following: since we are interested in the attitudes of the self-employed and how these differ from other groups, we include a dummy for being self-employed (as opposed to being full- or part-time employed, unemployed, housemaker, retired, in education, or other). We also include a measurement of respondents’ ideological orientation, measured as their self-placement on an integer 0–10 left–right scale, with higher values corresponding to a more conservative ideology. Finally, since we hypothesized that in Switzerland there are basic ideological differences between the linguistic regions, we include a dummy for living in the francophone region.

We control for the following other factors: gender (via a dummy for females), age, education (via a dummy for having completed upper secondary, upper vocational or university training), income (via a dummy for earning more than 8000 CHF/month), and political interest (measured on an integer 0–10 scale, with higher values corresponding to greater interest).

Before estimating regression models, we conducted a descriptive analysis to verify that there is meaningful variation in the ranks given to the different policy options in the data. The results are presented below. In addition, we verified that there really are overall significant differences in rankings assigned to each option using Friedman’s rank sum test as well as Wilcoxon signed rank tests for pairs of options. Both tests indicate that this is the case across both ranking tasks in both survey rounds.^4^

As a final note, we point out that the ‘raw’ coefficient estimates for respondent-specific variables generated by conditional logit models have no straightforward interpretation (they only indicate the change in choice probabilities of a given alternative *relative* to the omitted alternative). We therefore report the raw estimation results only in the supplementary materials and present more meaningful quantities (the marginal effects of predictors on predicted choice probabilities) here in the main text.

## Results

### Overall ranking of policy options

**Figure 1.**
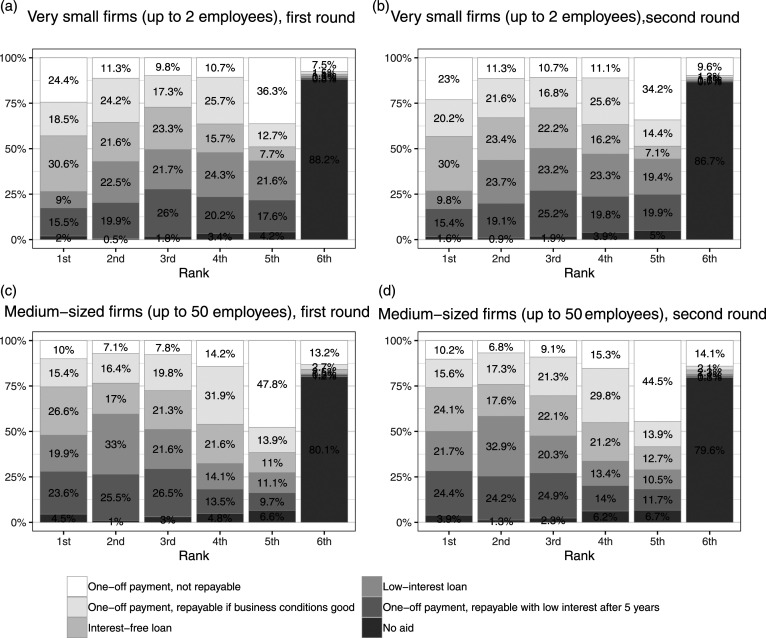
Ranking of policy preferences on state support for the self-employed, first round (April 2020) and second round (November 2020).

We start by presenting the overall rankings of policy options in both ranking tasks in each round in the four panels of Figure 1. The first striking result is the stability of the responses across the two rounds. The proportions of respondents who chose the various options barely changes between the two rounds, which is surprising given the fact that the time span covered by the two rounds was characterized by intensive public debate and policy activity on the issue at stake. While this applies of course only at the aggregate level (individual respondents or groups might have changed their attitudes), this absence of attitudinal change despite the very fluid contextual situation is worth pointing out.

Turning to the substantive results, it appears that: (a) an overwhelming majority of respondents ranks the option of giving no aid to small establishments as least desirable; and (b) that pluralities of respondents also rank the most generous option, a non-repayable payment, as next to last. This indicates that respondents do want small companies to receive some form of aid, but they are not enthusiastic about a payment to private businesses that will never have to be paid back.

We also find some differences between the two types of firms. For instance, the most generous option – the non-refundable payment – is considerably less popular in the case of larger companies than for small companies. It clearly ranks as the second-to-last alternative for medium-sized companies, while around a quarter of respondents place it in first place in the case of small firms. Conversely, the various types of repayable loan-based options are more commonly found in the upper ranks in the case of medium-sized companies. In brief, respondents are overall more generous toward very small than toward medium-sized companies. This is stable across the two survey rounds.

In the next step, we consider if there is variation between respondents in how they rank different policy options, as predicted by our hypotheses.

### Risk exposure or ideology?

Our first and second hypotheses concern the role of risk exposure versus ideology as determinants of support for financial help for the self-employed. We test them by looking at the position of the self-employed, that is, a group with high-risk exposure and anti-welfare ideology relative to the rest of the sample.

**Figure 2.**
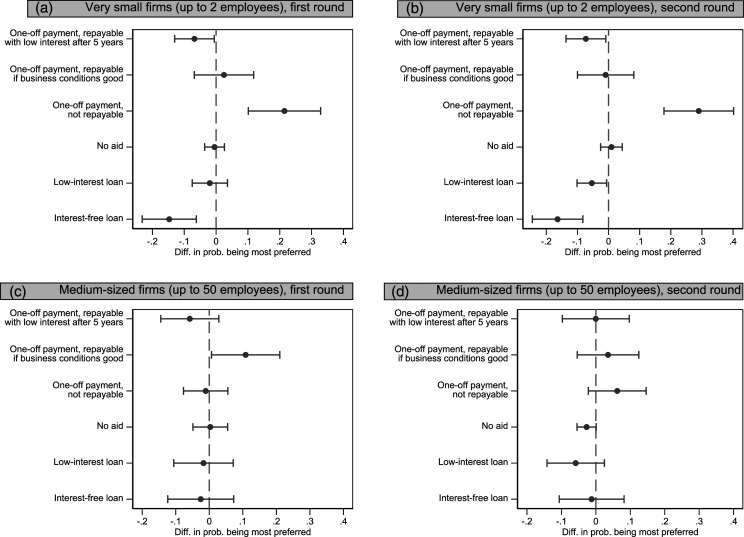
Preferred policy option (option ranked 1st) by employment status (self-employed vs all other statuses), first round (April 2020) and second round (November 2020).

Figure 2 displays the estimated marginal effects of being self-employed on the probability to prefer either of the different policy options for both types of firms. Starting with the case of very small companies (upper row), it is immediately apparent that there is a strong effect of being self-employed on preferring the most generous option, the non-repayable one-off payment. The self-employed prefer this option clearly and significantly more than other groups, and this is stable across the two survey rounds. Conversely, the self-employed are significantly less favourable toward two of the less generous options, interest-free loans or repayable payments. There are no differences between the self-employed and other respondents with respect to the remaining options. This finding is a strong indication that the self-employed person’s self-interest trumps their ideological predisposition toward limited government aid.

To substantiate this point further, we briefly look at the left–right orientation of the self-employed and how it changes between the two rounds of our survey (see also Figure S5 in the supplementary materials). A difference-of-means t-test reveals that the self-employed are initially (round 1) significantly more to the right than other respondents, but this difference turns statistically insignificant in round 2 (mean values in round 1 on the 0–10 scale, 5.63 vs 5.10, *p*-value = 0.03; round 2: 5.27 vs 5.09, *p*-value = 0.42). We take this as further confirmation that self-interest did override ideological convictions. In other words, the fact that the self-employed people became slightly more leftist over time suggests that they warmed up to the idea of more government involvement. This being said, we remain agnostic about how enduring this shift is given the earlier findings on the instability on shifts in attitudes in response to economic shocks by, for example, Margalit (2013).

When it comes to aid for larger firms of up to 50 employees, however, the preferences of the self-employed are no longer different from those of the rest of the population. The only significant effect we find here is that the self-employed are more supportive than others of giving larger companies a conditionally repayable transfer; this effect is not stable across the two survey rounds, however. Overall, help to large firms seems to be less polarizing than help to small firms, where we can see more disagreement within our sample.

### Solidarity from the left or the right?

Hypotheses 3 and 4 concern the impact of left–right positioning on help for the self-employed. The self-employed may receive support from pro-welfare voters who are found on the political left (H3) or rather from their typical allies on the right (H4). We argued that the direction of the association cannot be established on theoretical grounds alone, as we can expect both left-wing respondents and right-wing respondents to be more favourable to state support for the self-employed.

**Figure 3.**
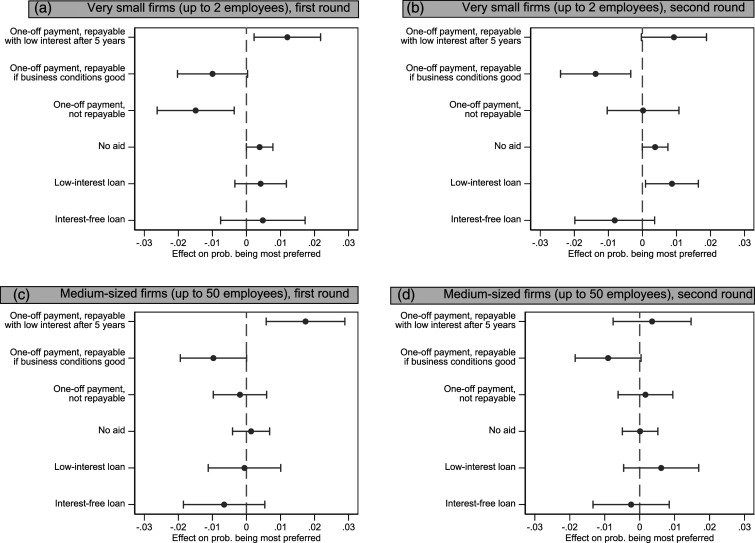
Preferred policy option (option ranked 1st) by position on the left–right axis (marginal effect of a more conservative ideology), first round (April 2020) and second round (November 2020).

When looking at the effects of the general ideological left–right orientation (Figure 3), our results show only a limited impact on policy preferences concerning help for the self-employed, and that this impact declines over time. With regard to very small firms in round 1, the option of a non-repayable payment is favoured by left-wing respondents. However, this effect of political orientation disappears completely in round 2, though it re-emerges for a slightly less favourable option: a one-off payment repayable if business conditions are good. Why this happened is unclear. It could be the result of some form of compassion fatigue among persons on the left induced by the fact that by November 2020 the costs of the various aid packages had reached impressive amounts, and they shifted to a less generous stance.

Preferences with regard to medium sized firms (up to 50 employees) are even less related to political orientation, with most effects being non-significant (the only exception being the one-off payment repayable with low interest after 5 years in panel C). In a way, as we move from small to slightly larger firms, the policy preferences of left-wing voters become more similar to those on the right, who have a clear preference for loans as opposed to non-refundable payments.

Thus, none of the two hypotheses on the role of political ideology finds clear confirmation. The effect of political orientation on the most favourable option found in round 1 disappeared in round 2. Since round 1 was carried out in an extremely exceptional and novel situation (which had somewhat normalized by round 2), suggests that we should not attempt generalizations of the political orientation effect observed in round 1.

It is also clear that the historical affinity between the self-employed and the parties on the right did not affect the policy preferences of right-wing voters in relation to financial support for the former. Additional models (not shown) focusing on voters of the main right-wing populist party (SVP-UDC), which in public discourse is very supportive of small businesses, do not contradict this finding. SVP-UDC voters have policy preferences that are indistinguishable from those of the rest of the sample. This is arguably related to the fact that this party intended to support the interests of small businesses not by advocating state help, but by demanding the removal of limitations to economic activity (for example, SVP, 2020).

## Discussion and conclusion

The self-employed are an underprotected group in most welfare states. This results from a well-established preference for self-reliance and the predominance of anti-statist values among this group. During the COVID-19 crisis, however, absence of social protection for the self-employed became a major policy problem. In this article, we were able to map public attitudes with regard to the most appropriate policy response.

First, risk exposure comes out clearly as the main determinant of attitudes toward state help for the self-employed. Respondents who identified themselves as self-employed were also those who supported the most generous options for small firms. Interestingly, this effect concerned only very small firms (up to two employees) and not larger ones (up to 50 employees). The historical association between self-employed status and opposition to state forms of social protection did not play out this time, suggesting that when anti-statist values and pro-welfare interests collide, the latter tend to prevail. This finding is in line with the view expressed by historians with regard to the change of attitude of the self-employed between the early postwar years and the 1960s and 1970s (Baldwin, 1990; Ferrera, 1993). The COVID-19 crisis is clearly different from a decade long economic decline, but the observed effect is rather similar, a strong demand for state protection among the self-employed in times of economic insecurity.

This result can also be interpreted as an indication of superiority of risk exposure to political values as a determinant of welfare attitudes (in line with Cusack et al., 2006; Häusermann et al., 2015). The self-employed included in our sample hold more right-wing views relative to employees, but were nonetheless more likely than the latter to favour generous state support. In addition, between round 1 and round 2, we notice a modest shift to the left of the average positioning of the self-employed in our sample. In round 2, the difference between them and employees is no longer significant. This result could indicate that after months of debate and frustration with right-wing parties’ opposition to non-repayable help, some self-employed workers shifted their position to the left as if, with time, political values adjusted to self-interest.

Second, political orientation did not count much as a determinant of policy preference. Only at the very beginning of the health crisis do we find a stronger preference for the most generous form of help among left-wing respondents, but this effect disappeared a few months later. The sudden onset of the crisis and the adoption of the lockdown created a very unusual situation, and results observed at that time are particularly difficult to generalize. As a result, we give more weight to the findings from the second round of our survey carried out after several months into the pandemic. The absence of a clear association between positioning on the left–right axis and attitudes towards support for the self-employed may result from lack of dependency between two measurements or the presence of contrasting effects, since both voters on the left and on the right have reasons to favour help for the self-employed. In this case, however, we would observe a U-shaped relationship between left–right positioning and support, which we don’t (see Figure S10 in the supplementary materials). On the basis of our data, lack of dependency seems the most likely explanation.

Our study also shows a clear difference depending on the size of the firm. This was not necessarily something we expected. One possible interpretation is that when asked about a firm with some 50 employees, respondents may think ‘firm’ rather than ‘person’ and possibly attitudes towards help to firms depend on a reasoning that is totally different from perceptions of deservingness to social benefits. In contrast, when asked about help to a firm with up to two employees, respondents think of the owner, that is, a person, and then the well-known mechanisms that determine solidarity play out.

The COVID-19 crisis created a totally new and unexpected situation for all of us. For many self-employed people it resulted in the total or nearly total inability to obtain an income from the market. Given their underprotected status in the welfare state, their situation was suddenly one of extreme vulnerability. Our study shows that society reacted in an overall solidaristic fashion, as the option of ‘no aid’ was clearly the least preferred. The self-employed reacted too. Interests prevailed over political values, as on this occasion they were considerably more likely than the rest of the sample to support the most generous forms of help for small business. In addition, our survey provides some indication that their overall political position may have shifted leftwards after some 7 months into the crisis.

The COVID-19 pandemic highlighted the extreme vulnerability of groups that depend primarily on market income and without access to safety nets. In the short term, the result was, for the self-employed, a reorientation of preferences in the direction of more support for inclusion in redistributive arrangements. Will this experience change attitudes in the long run too? This is not inconceivable, since historically, major events have shifted public opinion and the COVID-19 pandemic could have raised the awareness of the risky nature of dependence on markets among many self-employed people. To find out whether this is indeed the case, is a task for future research.

## Supplemental Material

Supplemental Material - Sometimes needs change minds: Interests and values as determinants of attitudes towards state support for the self-employed during the COVID-19 crisis Click here for additional data file. Supplemental Material for Sometimes needs change minds: Interests and values as determinants of attitudes towards state support for the self-employed during the COVID-19 crisis by Giuliano Bonoli, Flavia Fossati, Mia Gandenberger and Carlo Knotz in Journal of European Social Policy

## Notes

1. Source: https://www.bfs.admin.ch/bfs/fr/home/statistiques/travail-remuneration/enquetes/espa/publications-resultats.assetdetail.20544323.html

2. This corresponds to the *independence of irrelevant alternatives* (IIA) assumption (Train, 2009: 45–7).

3. We test for this, following Allison and Christakis (1994), by estimating models that include intercepts for *J-1* alternatives (the standard baseline model) plus interaction terms with a dummy for upper ranks (for each respondent, those alternatives which they had ranked as the top three) and then testing for the joint significance of the interaction terms via a likelihood-ratio test. These tests indicate greater randomness for lower ranks across both ranking tasks, even when we apply a more restrictive coding and consider only the two first ranked alternatives to be ‘upper ranks’.

4. The rank sum test evaluates whether some options are consistently higher or lower ranked than others, and in our case strongly rejects the null hypothesis that no options are consistently different for both ranking tasks. The Wilcoxon signed rank test evaluates whether there are significant differences in the ranks given between pairs of options. We test for differences between options with adjacent average ranks and find generally significant differences, but less so in the case of medium-sized companies. We provide the results of these tests in the supplementary materials.

## References

[bibr1-09589287221106977] Abraham KG Haltiwanger J Sandusky K , et al. (2019) The rise of the gig economy: fact or fiction? AEA Papers and Proceedings 109: 357–361.

[bibr2-09589287221106977] Allison PD Christakis NA (1994) Logit models for sets of ranked items. Sociological Methodology 24: 199–228.

[bibr3-09589287221106977] Baldwin P (1990) The Politics of Social Solidarity: Class Bases of the European Welfare State 1875–1975. Cambridge: Cambridge University Press.

[bibr4-09589287221106977] Baptista I Marlier E Spasova S , et al. (2021) Social Protection and Inclusion Policy Responses to the COVID-19 Crisis: An Analysis of Policies in 35 Countries. Brussels: European Commission: European Social Policy Network (ESPN).

[bibr5-09589287221106977] Barisone M De Luca D (2018) Do the self-employed still vote for centre-right parties? The cases of the UK, Italy and Spain. Electoral Studies 52: 84–93.

[bibr6-09589287221106977] Blekesaune M (2007) Economic conditions and public attitudes to welfare policies. European Sociological Review 23(3): 393–403.

[bibr7-09589287221106977] Blumenau J Hicks T Jacobs AM , et al. (2021) Testing negative: the non-consequences of COVID-19 on mass political attitudes. Paper Presented at the Max Planck Online Workshop in Comparative Political Economy (18 February 2021).

[bibr35-09589287221106977] Buschoff KS Schmidt C (2009) Adapting labour law and social security to the needs of the ‘new self-employed’: comparing the UK, Germany and the Netherlands. Journal of European Social Policy 19(2): 147–159.

[bibr8-09589287221106977] Codagnone C Lupiáñez-Villanueva F Tornese P , et al. (2018) Behavioural Study on the Effects of an Extension of Access to Social Protection for People in All Forms of Employment. Luxembourg: Publications Office of the European Union.

[bibr9-09589287221106977] Crettaz E (2013) A state-of-the-art review of working poverty in advanced economies: theoretical models, measurement issues and risk groups. Journal of European Social Policy 23(4): 347–362.

[bibr10-09589287221106977] Cusack T Iversen T Rehm P (2006) Risks at work: the demand and supply sides of government redistribution. Oxford Review of Economic Policy 22(3): 365–389.

[bibr11-09589287221106977] Emmenegger P (2009) Barriers to entry: insider/outsider politics and the political determinants of job security regulations. Journal of European Social Policy 19(2): 131–146.

[bibr12-09589287221106977] Ferrera M (1993) Modelli di solidarietà: Politica e riforme sociali nelle democrazie. Bologna: Il Mulino.

[bibr13-09589287221106977] Halleröd B Ekbrand H Bengtsson M (2015) In-work poverty and labour market trajectories: poverty risks among the working population in 22 European countries. Journal of European Social Policy 25(5): 473–488.

[bibr14-09589287221106977] Häusermann S (2012) The politics of new and old social risks. In Bonoli G Natali D (eds) The Politics of the New Welfare State. Oxford: Oxford University Press, 111–134.

[bibr15-09589287221106977] Häusermann S Kurer T Schwander H (2015) High skilled outsiders? Labor market vulnerability, education and welfare state preferences. Socio Economic Review 13(2): 235–258.

[bibr16-09589287221106977] Huber E Stephens J (2001) Development and Crisis of the Welfare State: Parties and Policies in the Global Markets. Chicago, IL: The University of Chicago Press.

[bibr17-09589287221106977] Huber E Stephens J (2006) Combatting old and new social risks. In Armingeon K Bonoli G (eds) The Politics of Postindustrial Welfare States. London: Routledge, 143–155.

[bibr18-09589287221106977] Iversen T Soskice D (2001) An asset theory of social policy preferences. American Political Science Review 95(4): 875–895.

[bibr19-09589287221106977] Jansen G (2016a) Farewell to the rightist self-employed? ‘New self-employment’ and political alignments. Acta Politica 52(3): 306–338.

[bibr20-09589287221106977] Jansen G (2016b) Self-employment as atypical or autonomous work: diverging effects on political orientations. Socio-Economic Review 17(2): 381–407.

[bibr21-09589287221106977] Korpi W (1983) The Democratic Class Struggle. London: Routledge & Kegan Paul.

[bibr22-09589287221106977] Mares I (2003) The Politics of Social Risk: Business and Welfare State Development. Cambridge: Cambridge University Press.

[bibr23-09589287221106977] Margalit Y (2013) Explaining social policy preferences: evidence from the Great Recession. American Political Science Review 107(1): 80–103.

[bibr24-09589287221106977] Mau S (2003) The Moral Economy of the Welfare State: Britain and Germany Compared. Abingdon UK: Routledge.

[bibr25-09589287221106977] McFadden D (1974) Conditional logit analysis of qualitative choice behavior. In Zarembka P (ed) Frontiers in Econometrics. Cambridge, MA: Academic Press.

[bibr26-09589287221106977] Meuleman B Roosma F Abts K (2020) Welfare deservingness opinions from heuristic to measurable concept: the CARIN deservingness principles scale. Social Science Research 85: 102352, DOI: 10.1016/j.ssresearch.2019.102352. 31789191

[bibr27-09589287221106977] OECD (2020) Supporting People and Companies to Deal with the COVID-19 Virus: Options for an Immediate Employment and Social-Policy Response. Paris: OECD.

[bibr28-09589287221106977] Oesch D (2006) Redrawing the Class Map: Stratification and Institutions in Britain, Germany, Sweden and Switzerland. London: Palgrave.

[bibr29-09589287221106977] Paster T (2013) Business and welfare state development: why did employers accept social reforms? World Politics 65(3): 416–451.

[bibr30-09589287221106977] Rehm P (2009) Risks and redistribution: an individual-level analysis. Comparative Political Studies 42(7): 855–881.

[bibr31-09589287221106977] Rehm P (2016) Risk Inequality and Welfare States: Social Policy Preferences, Development, and Dynamics. Cambridge: Cambridge University Press.

[bibr32-09589287221106977] Rossetti F Abts K Meuleman B , et al. (2020) ‘First the grub, then the morals’? Disentangling the self-interest and ideological drivers of attitudes towards demanding activation policies in Belgium. Journal of Social Policy 50(2): 346–366.

[bibr33-09589287221106977] Sabat I Neuman-Böhme S Varghese NE , et al. (2020) United but divided: policy responses and people’s perceptions in the EU during the COVID-19 outbreak. Health Policy 124(9): 909–918. 32631613 10.1016/j.healthpol.2020.06.009 PMC7307992

[bibr34-09589287221106977] Scase R Goffee R (1981) Traditional petty bourgeois attitudes: the case of self-employed craftsmen. The Sociological Review 29(4): 729–747.

[bibr36-09589287221106977] Shin Y-K Kemppainen T Kuitto K (2020) Precarious work, unemployment benefit generosity and universal basic income preferences: a multilevel study on 21 European countries. Journal of Social Policy 50(2): 323–345.

[bibr37-09589287221106977] Spasova S Bouget D Ghailani D , et al. (2017) Access to Social Protection for People Working on Non-standard Contracts and as Self-Employed in Europe. Brussels: European Commission: European Social Policy Network (ESPN).

[bibr38-09589287221106977] Svallfors S (1995) The end of class politics? Structural cleavages and attitudes to Swedish welfare policies. Acta Sociologica 38(1): 53–74.

[bibr39-09589287221106977] Svallfors S (2004) Class, attitudes and the welfare state: Sweden in comparative perspective. Social Policy & Administration 38(2): 119–138.

[bibr40-09589287221106977] SVP (Schweizerische Volkspartei) (2020) Es darf keinen zweiten Lockdown geben. Medienmitteilung, 28 October 2020.

[bibr41-09589287221106977] Tóth IG Fondeville N Herke B , et al. (2020) Attitudes Towards Adequacy and Sustainability of Social Protection Systems in the EU. Brussels: European Commission.

[bibr42-09589287221106977] Train KE (2009) Discrete Choice Methods with Simulation. Cambridge: Cambridge University Press.

[bibr43-09589287221106977] van Oorschot W Roosma F Meuleman B , et al. (eds) (2017) The Social Legitimacy of Targeted Welfare: Attitudes to Welfare Deservingness. Cheltenham and Northampton, MA: Edward Elgar Publishing.

[bibr44-09589287221106977] Wehl N (2019) The (ir)relevance of unemployment for labour market policy attitudes and welfare state attitudes. European Journal of Political Research 58(1): 141–162.

