# Supplementary material for: Sometimes needs change minds: Interests and values as determinants of attitudes towards state support for the self-employed during the COVID-19 crisis
Source: J Eur Soc Policy. 2022 Jul 20;32(4):407–21. doi: 10.1177/09589287221106977 (PMC9301353; doi:10.1177/09589287221106977)
Supplement: Supplemental Material - Sometimes needs change minds: Interests and values as determinants of attitudes towards state support for the self-employed during the COVID-19 crisis [file sj-pdf-1-esp-10.1177_09589287221106977.pdf]

Supplementary material

Table S1: State help for SMEs and the self-employed during the COVID crisis in Switzerland – chronology of the main decisions

**25.03.2020**	Federal government adopts an emergency programme for SMEs, whereby they can access loans guaranteed by the federal government. These are interest free for amounts up to 500,000 CHF. Above this amount, an annual interest of 0.5% is charged. Total amount committed 20 billion CHF.<br>Adoption of new income replacement scheme for the self-employed, similar to temporary employment benefit.
**03.04.2020**	Increase to 40 billion CHF of the amount committed to support SMEs.
**25.11.2020**	Faced with the second wave of the pandemic and after deciding a second partial lockdown, the Federal government adopts a new instrument “aid for hardship cases” consisting of non-repayable lump sums. The scheme is meant to be co-financed by the Federal government and the Cantons. Amount committed 1 billion CHF. Hardship cases are defined as firms with a decline of at least 40% of their turnover.
**13.01.2021**	The Federal government increases the funds available for the “hardship cases” programme and reduces the requirements to be eligible for the programme. Firms which were closed for at least 40 days after 1.11.2020 are now eligible for it, without needed to show a reduction in turnover of at least 40%.
**27.01.2021**	The Federal government further increases the funds available for the “hardship cases” programme to a total of 5 billion CHF.
**17.01.2021**	Increase to up to 10 billion CHF the of the Federal contribution to the “hardship cases” programme.

Source: https://covid19.easygov.swiss/fr/#anchor-7, visited 22.04.2021

Figure S1: Age distribution of survey samples and comparison with official statistics

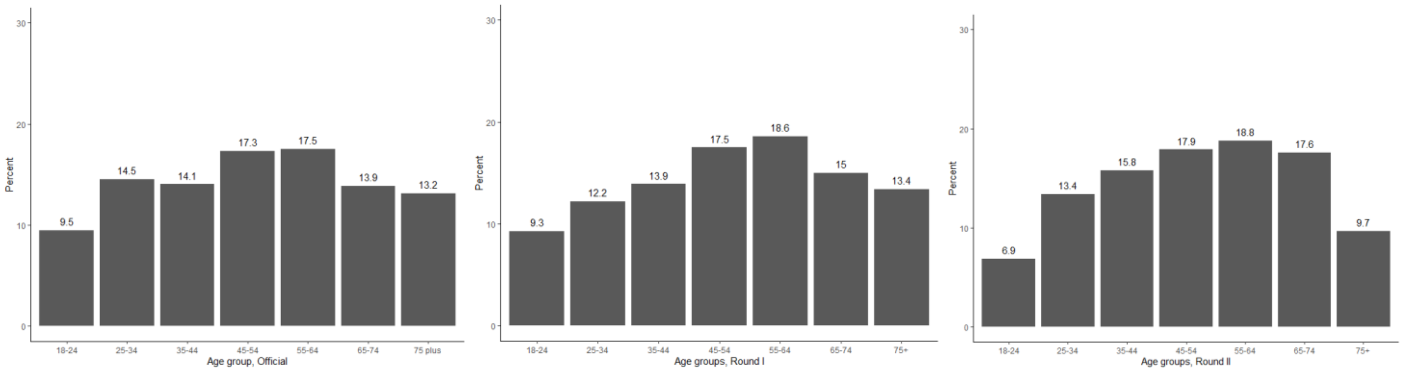

Notes: Official figures based on 2019 STATPOP data of the Swiss Federal Office for Statistics: https://www.bfs.admin.ch/bfs/de/home/statistiken/bevoelkerung/stand-entwicklung/alter-zivilstand-staatsangehoerigkeit.assetdetail.13707177.html, last access on 11 January 2021, official figures computed for population aged 18 and older.

Figure S2: Gender distribution of survey samples and comparison with official statistics

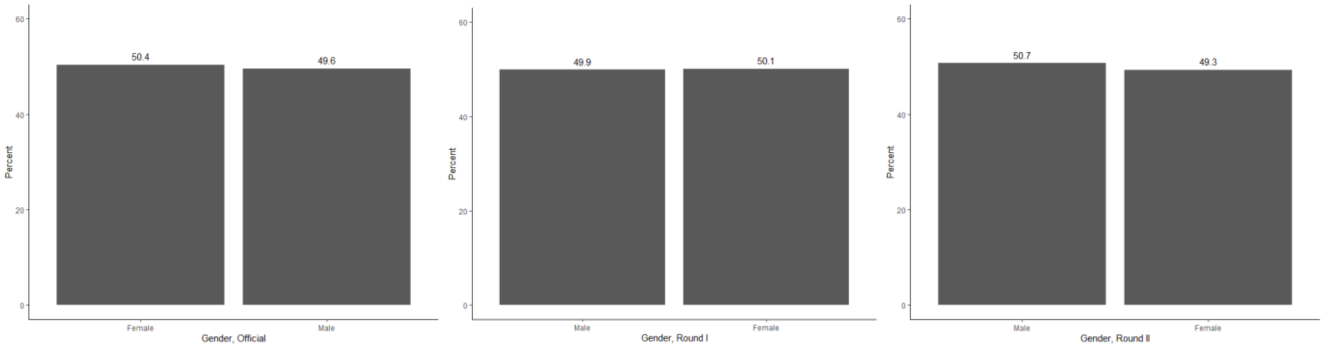

Notes: Official figures based on 2019 STATPOP data of the Swiss Federal Office for Statistics: https://www.bfs.admin.ch/bfs/de/home/statistiken/bevoelkerung/stand-entwicklung/bevoelkerung.html, last access on 11 January 2021, official figures computed for population aged 18 and older. We screened out respondents who indicated that they did not want to reveal their gender at the beginning of the survey.

Figure S3: Distribution of respondents over linguistic regions and comparison with official statistics

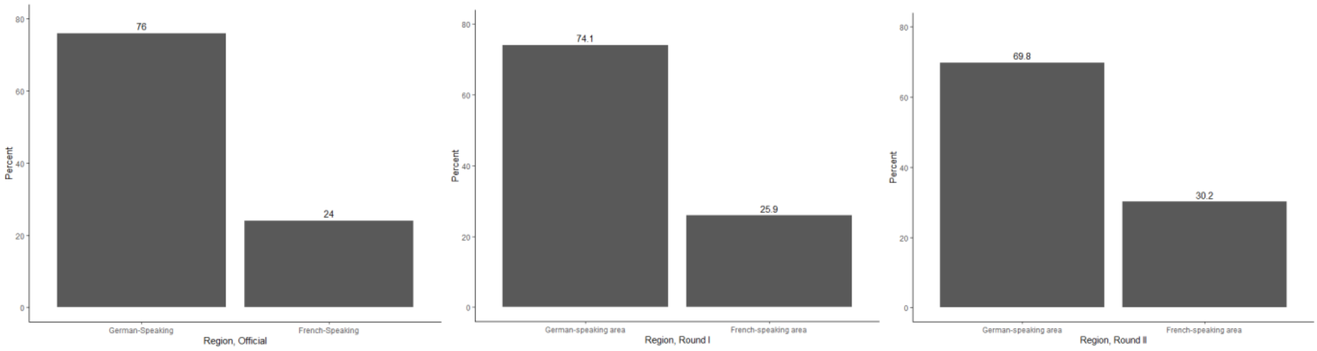

Notes: Offical figures based on 2018STATPOP data of the Swiss Federal Office for Statistics: https://www.bfs.admin.ch/bfs/de/home/statistiken/bevoelkerung/stand-entwicklung.assetdetail.13707332.html, last access on 11 January 2021, official figures computed for population aged 18 and older; Ticino (4%) not considered.

Figure S4: Distribution of educational attainment within survey samples and comparison with official statistics

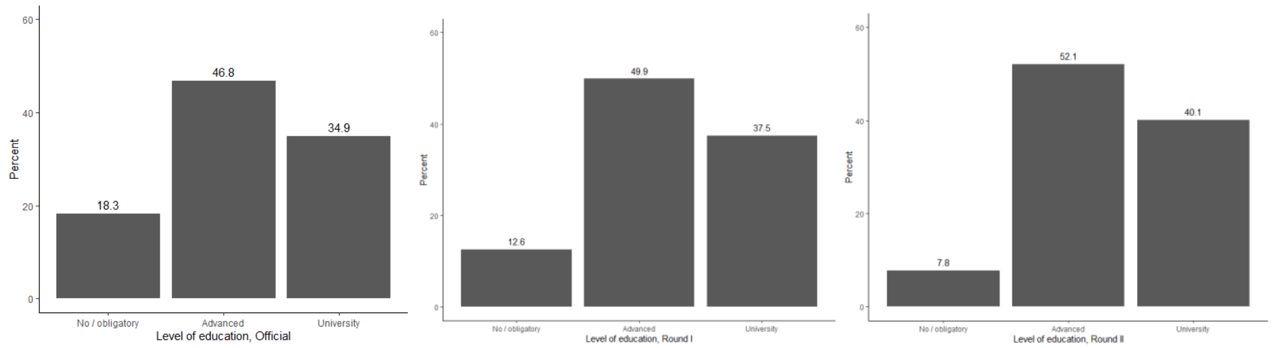

Notes: Official figures based on Schweizerische Arbeitskräfteerhebung (SAKE) data from 2018 of the Swiss Federal Office for Statistics: https://www.bfs.admin.ch/bfs/de/home/statistiken/bevoelkerung/migration-integration/integrationindikatoren/indikatoren/abgeschlossene-ausbildung.assetdetail.14876535.html last access on 11 January 2021, official figures computed for population aged 18 and older.

Figure S5: Left-right self-placement of the self-employed compared to other respondents

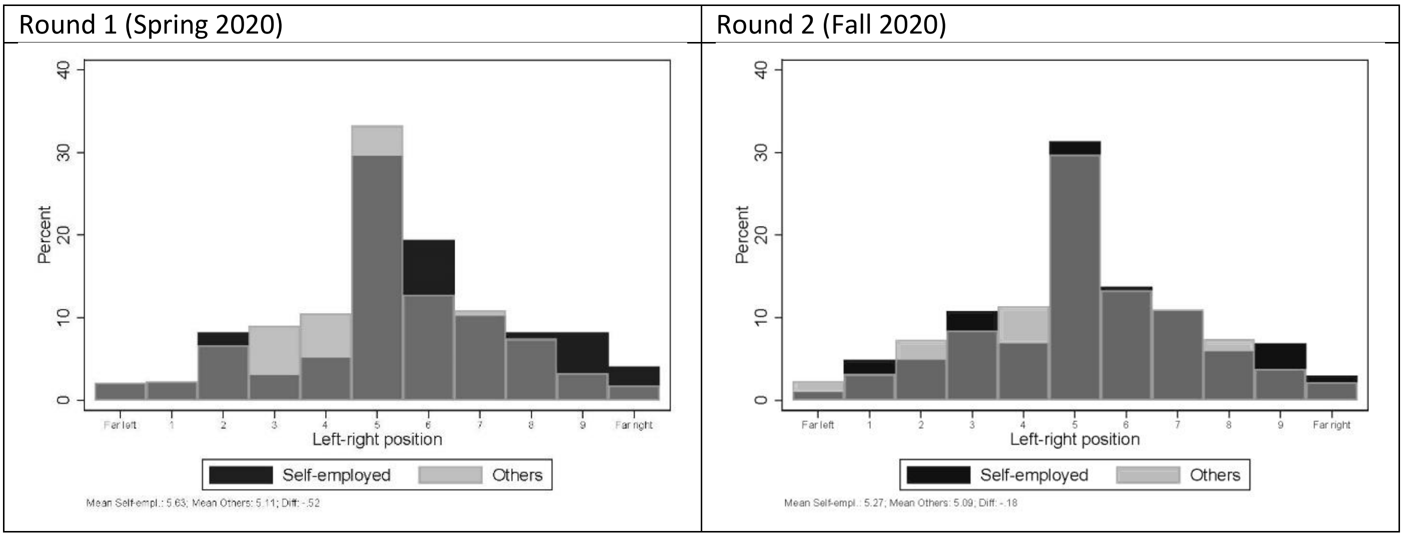

Figure S6: Gender of the self-employed compared to other respondents

Round 1 (Spring 2020)	Round 2 (Fall 2020)
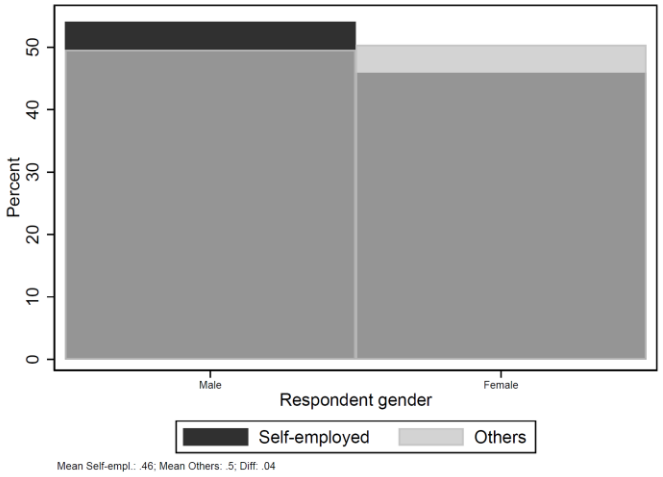	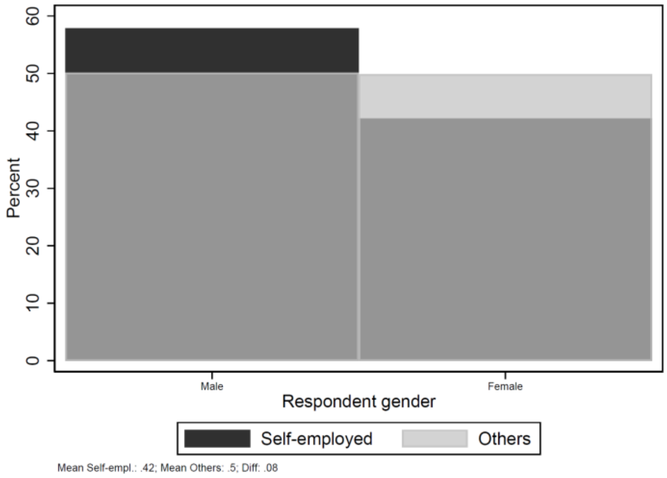

Figure S7: Education level of the self-employed compared to other respondents

Round 1 (Spring 2020)	Round 2 (Fall 2020)
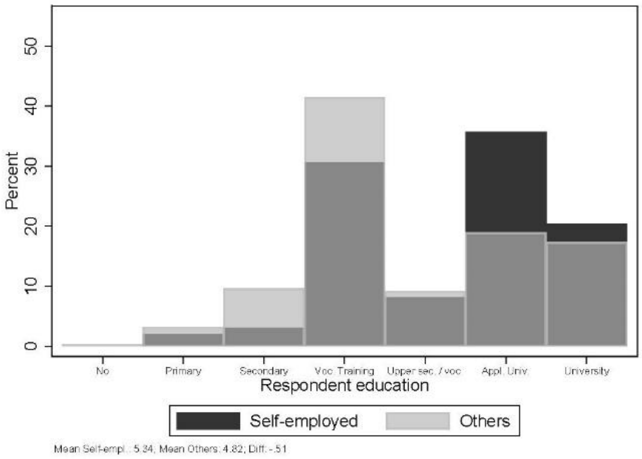	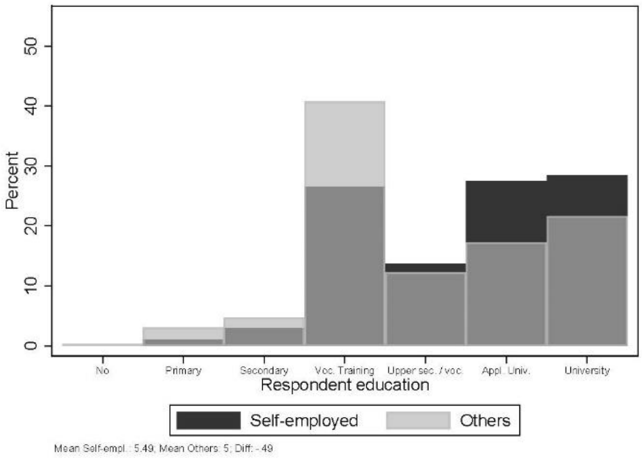

Figure S8: Age of the self-employed compared to other respondents

Round 1 (Spring 2020)	Round 2 (Fall 2020)
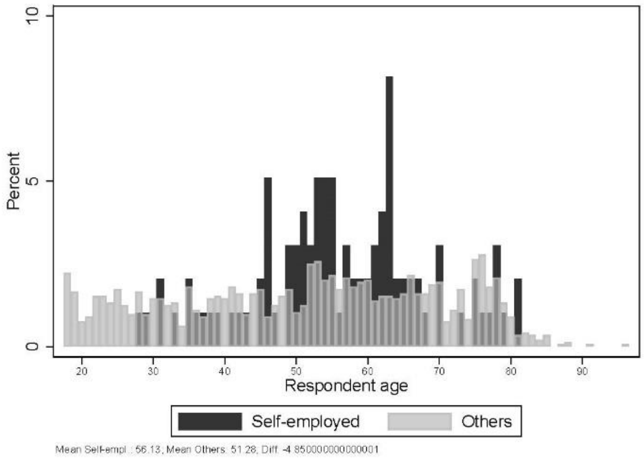	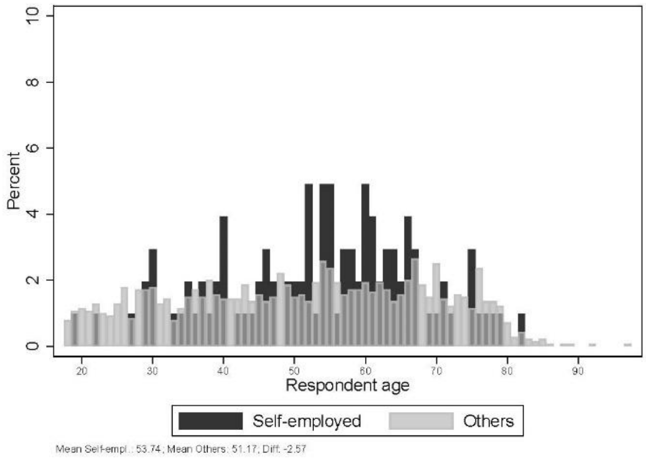

Figure S9: Respondents in employment by work status

Round 1 (Spring 2020)	Round 2 (Fall 2020)
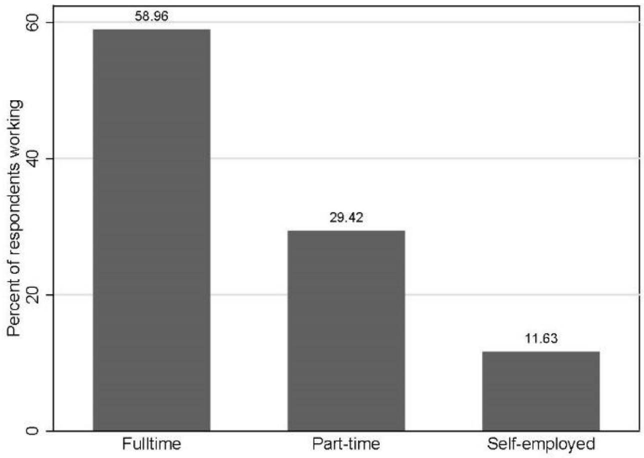	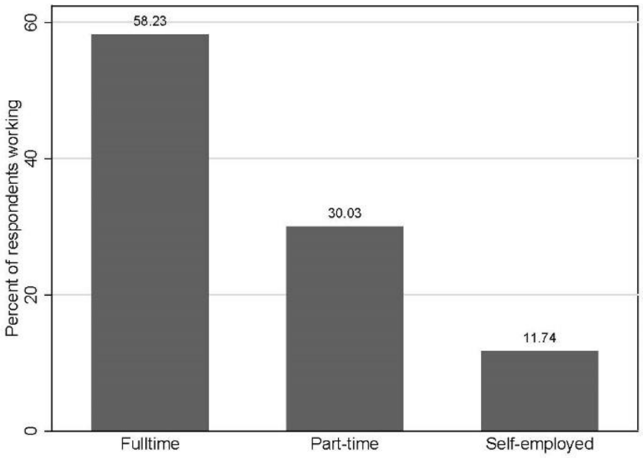

Figure S10: Preferred policy option (option ranked 1st) by position on the left-right axis,

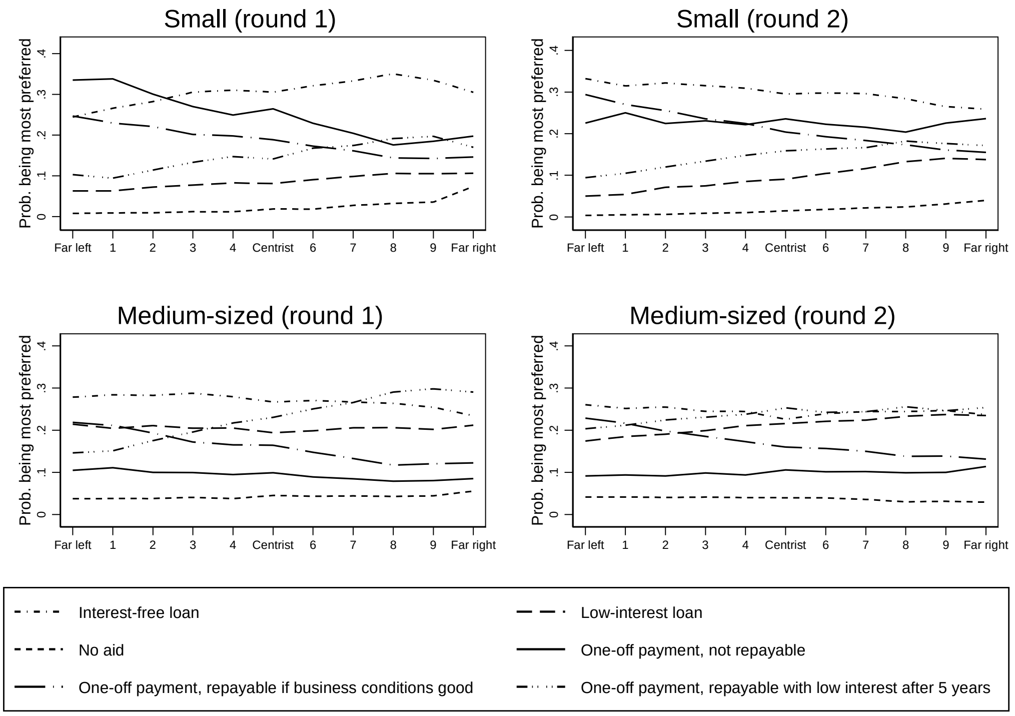

Table S2: Results of Friedman rank sum tests

	χ^2	Degrees of freedom	*p*
Small companies			
Round 1	2822.4	5	<0.001
Round 2	2719.3	5	<0.001
Medium-sized companies			
Round 1	2824.0	5	<0.001
Round 2	2750.9	5	<0.001

Table S3: Results of Wilcoxon rank tests (small companies, first round)

	Comparison	V	p
1	Interest-free loan vs. one-off payment, repayable if business conditions good	459100	<0.001
2	One-off payment, repayable if business conditions good vs. one-off payment, repayable after five years	549848	0.021
3	One-off payment, repayable after five years vs. low-interest loan	501306	<0.001
4	Low-interest loan vs. one-off payment, not repayable	559528	0.083
5	One-off payment, not repayable vs. no aid	96584	<0.001

Table S4: Results of Wilcox rank tests (medium-sized companies, first round)

	Comparison	V	p
1	One-off payment, repayable after five years vs. low-interest loan	585680	0.832
2	Low-interest loan vs. interest-free loan	530897	0.001
3	Interest-free loan vs. one-off payment, repayable if business conditions good	471356	<0.001
4	One-off payment, repayable if business conditions good vs. one-off payment, not repayable	265331	<0.001
5	One-off payment, not repayable vs. no aid	231940	<0.001

Table S5: Results of Wilcoxon rank tests (small companies, second round)

	Comparison	V	p
1	Interest-free loan vs. one-off payment, repayable if business conditions good	427234	<0.001
2	One-off payment, repayable if business conditions good vs. one-off payments, repayable after five years	517260	0.008
3	One-off payments, repayable after five years vs. low-interest loan	525739	0.028
4	Low-interest loan vs. one-off payment, not repayable	497476	<0.001
5	One-off payment, not repayable vs. no aid	102045	<0.001

Table S6: Results of Wilcoxon rank tests (medium-sized companies, second round)

	Comparison	V	p
1	One-off payment, repayable after five years vs. low-interest loan	581358	0.218
2	Low-interest loan vs. interest-free loan	478624	<0.001
3	Interest-free loan vs. one-off payment, repayable if business conditions good	481165	<0.001
4	One-off payment, repayable if business conditions good vs. one-off payment, not	249369	<0.001

	repayable		
5	One-off payment, not repayable vs. no aid	218782	<0.001

Table S7: Detailed estimation results (medium-sized companies, first round)

	Model 1		Model 2	
Low interest loan				
Self-employed	0.11	(0.37)	0.01	(0.03)
French	-0.23	(-1.31)	-0.05	(-0.28)
Left-right	0.00	(0.05)	0.02	(0.53)
Gender			0.24	(1.35)
Age			-0.00	(-0.10)
Education			-0.14	(-0.81)
Pol. interest			0.07*	(2.10)
Income			-0.24	(-1.24)
Constant	-0.25	(-1.18)	-0.80*	(-2.01)
No aid				
Self-employed	-0.35	(-0.56)	0.17	(0.27)
French	0.08	(0.28)	0.26	(0.81)
Left-right	-0.01	(-0.14)	0.06	(0.77)
Gender			0.02	(0.06)
Age			-0.01	(-1.14)
Education			-0.68*	(-2.07)
Pol. interest			0.02	(0.41)
Income			-0.57	(-1.46)
Constant	-1.74*	(-4.85)	-1.45*	(-2.14)
One off payment, not repayable				
Self-employed	-0.04	(-0.10)	-0.01	(-0.03)
French	0.12	(0.57)	0.38	(1.66)
Left-right	-0.01	(-0.23)	0.00	(0.05)
Gender			0.40	(1.78)
Age			-0.00	(-0.56)
Education			-0.25	(-1.07)
Pol. interest			-0.00	(-0.02)
Income			-0.21	(-0.82)
Constant	-0.95*	(-3.66)	-1.02*	(-2.02)
One off payment, repayable if business conditions good				
Self-employed	0.44	(1.41)	0.65	(1.91)
French	-0.02	(-0.11)	0.10	(0.50)
Left-right	-0.07	(-1.78)	-0.04	(-0.88)
Gender			0.43*	(2.27)
Age			-0.00	(-0.13)
Education			-0.30	(-1.55)
Pol. interest			0.03	(0.70)
Income			-0.32	(-1.45)
Constant	-0.22	(-0.99)	-0.53	(-1.24)
One off payment, repayable with low interest after 5 years				
Self-employed	-0.22	(-0.71)	-0.19	(-0.52)
French	-0.50*	(-2.91)	-0.47*	(-2.41)
Left-right	0.07*	(2.05)	0.10*	(2.50)
Gender			0.14	(0.81)
Age			0.00	(0.36)
Education			-0.06	(-0.37)
Pol. interest			0.02	(0.70)
Income			-0.28	(-1.46)
Constant	-0.37	(-1.81)	-0.74	(-1.93)
Observations	9210		7440	

Respondents	1535		1240	
Model p-value (chi-sq.)	0.015	(29.21)	0.031	(58.23)

z statistics in parentheses
* $p < 0.05$

Table S8: Detailed estimation results (small companies, first round)

	Model 1		Model 2	
Low interest loan				
Self-employed	0.25	(0.58)	0.35	(0.70)
French	-0.40	(-1.73)	-0.28	(-1.05)
Left-right	0.01	(0.26)	0.03	(0.62)
Gender			0.19	(0.82)
Age			-0.00	(-0.12)
Education			-0.16	(-0.70)
Pol. interest			0.06	(1.36)
Income			-0.03	(-0.11)
Constant	-1.21*	(-4.36)	-1.76*	(-3.35)
No aid				
Self-employed	-0.42	(-0.40)	0.38	(0.35)
French	0.49	(1.26)	0.98*	(2.32)
Left-right	0.20*	(2.14)	0.18	(1.76)
Gender			-0.14	(-0.31)
Age			-0.00	(-0.13)
Education			-1.28*	(-2.54)
Pol. interest			0.07	(0.82)
Income			-0.62	(-1.06)
Constant	-4.02*	(-6.71)	-3.81*	(-3.81)
One off payment, not repayable				
Self-employed	0.96*	(3.42)	1.32*	(3.97)
French	0.11	(0.74)	0.42*	(2.43)
Left-right	-0.08*	(-2.37)	-0.08*	(-2.14)
Gender			0.51*	(3.08)
Age			-0.00	(-1.03)
Education			-0.49*	(-2.93)
Pol. interest			0.01	(0.35)
Income			-0.29	(-1.50)
Constant	0.08	(0.43)	0.22	(0.60)
One off payment, repayable if business conditions good				
Self-employed	0.36	(1.06)	0.78*	(2.07)
French	-0.11	(-0.64)	0.04	(0.23)
Left-right	-0.09*	(-2.42)	-0.07	(-1.78)
Gender			0.65*	(3.69)
Age			-0.00	(-0.30)
Education			-0.49*	(-2.71)
Pol. interest			0.06	(1.65)
Income			-0.07	(-0.34)
Constant	-0.04	(-0.19)	-0.54	(-1.34)
One off payment, repayable with low interest after 5 years				
Self-employed	0.07	(0.19)	0.03	(0.06)
French	-0.91*	(-4.24)	-0.90*	(-3.61)
Left-right	0.04	(1.02)	0.07	(1.46)
Gender			0.45*	(2.36)
Age			-0.01	(-0.97)
Education			-0.03	(-0.17)
Pol. interest			0.07	(1.85)
Income			-0.13	(-0.64)
Constant	-0.72*	(-3.09)	-1.23*	(-2.84)
Observations	9210		7440	
Respondents	1535		1240	
Model p-value (chi-sq.)	0.000	(62.78)	0.000	(119.16)

zt statistics in parentheses
* $p < 0.05$

Table S9: Detailed estimation results (medium-sized companies, second round)

	Model 1		Model 2	
Low interest loan				
Self-employed	-0.20	(-0.62)	-0.26	(-0.70)
French	-0.18	(-1.10)	-0.29	(-1.59)
Left-right	0.02	(0.46)	0.04	(1.00)
Gender			0.05	(0.29)
Age			0.01	(1.08)
Education			0.02	(0.13)
Pol. interest			-0.05	(-1.55)
Income			0.01	(0.06)
Constant	-0.12	(-0.58)	-0.19	(-0.47)
No aid				
Self-employed	-0.71	(-0.95)	-1.07	(-1.03)
French	-0.40	(-1.26)	-0.33	(-0.96)
Left-right	0.02	(0.27)	0.01	(0.18)
Gender			0.17	(0.52)
Age			-0.02*	(-1.96)
Education			0.38	(1.11)
Pol. interest			-0.12	(-1.88)
Income			-0.51	(-1.37)
Constant	-1.75*	(-4.58)	-0.32	(-0.43)
One off payment, not repayable				
Self-employed	0.49	(1.46)	0.55	(1.42)
French	-0.02	(-0.09)	-0.03	(-0.15)
Left-right	-0.00	(-0.01)	0.03	(0.54)
Gender			-0.13	(-0.60)
Age			-0.00	(-0.19)
Education			-0.31	(-1.34)
Pol. interest			-0.09*	(-2.03)
Income			-0.05	(-0.22)
Constant	-0.89*	(-3.43)	-0.20	(-0.39)
One off payment, repayable if business conditions good				
Self-employed	0.34	(1.07)	0.25	(0.71)
French	0.22	(1.25)	0.22	(1.20)
Left-right	-0.03	(-0.82)	-0.05	(-1.08)
Gender			-0.05	(-0.28)
Age			-0.00	(-0.17)
Education			-0.02	(-0.11)
Pol. interest			-0.03	(-0.93)
Income			-0.11	(-0.53)
Constant	-0.38	(-1.71)	0.09	(0.20)
One off payment, repayable with low interest after 5 years				
Self-employed	-0.16	(-0.53)	0.05	(0.14)
French	-0.67*	(-3.91)	-0.66*	(-3.53)
Left-right	0.02	(0.47)	0.03	(0.68)
Gender			0.17	(0.96)
Age			0.01	(1.37)
Education			-0.21	(-1.21)
Pol. interest			-0.04	(-1.19)
Income			0.00	(0.02)
Constant	0.12	(0.58)	-0.01	(-0.02)
Observations	8988		7530	
Respondents	1498		1255	
Model p-value (chi-sq.)	0.002	(35.94)	0.014	(62.20)

z statistics in parentheses
* $p < 0.05$

Table S10: Detailed estimation results (small companies, second round)

	Model 1		Model 2	
Low interest loan				
Self-employed	-0.59	(-1.06)	-0.04	(-0.07)
French	-0.76^{*}	(-3.31)	-0.75^{*}	(-2.91)
Left-right	0.09	(1.88)	0.12^{*}	(2.40)
Gender			0.01	(0.05)
Age			0.01	(1.48)
Education			0.07	(0.31)
Pol. interest			0.07	(1.49)
Income			-0.36	(-1.47)
Constant	-1.34^{*}	(-5.02)	-2.46^{*}	(-4.58)
No aid				
Self-employed	0.57	(0.73)	1.23	(1.51)
French	-0.48	(-0.99)	-0.25	(-0.49)
Left-right	0.21^{*}	(2.08)	0.27^{*}	(2.30)
Gender			-0.09	(-0.19)
Age			-0.00	(-0.30)
Education			0.18	(0.36)
Pol. interest			-0.07	(-0.78)
Income			-0.40	(-0.75)
Constant	-3.99^{*}	(-6.21)	-3.70^{*}	(-3.29)
One off payment, not repayable				
Self-employed	1.17^{*}	(4.26)	1.65^{*}	(4.78)
French	0.03	(0.22)	0.16	(0.97)
Left-right	0.02	(0.45)	0.03	(0.74)
Gender			0.37^{*}	(2.23)
Age			0.00	(0.53)
Education			-0.03	(-0.17)
Pol. interest			-0.01	(-0.28)
Income			-0.44^{*}	(-2.37)
Constant	-0.45^{*}	(-2.33)	-0.70	(-1.80)
One off payment, repayable if business conditions good				
Self-employed	0.32	(0.97)	0.71	(1.82)
French	-0.04	(-0.27)	0.03	(0.15)
Left-right	-0.04	(-1.21)	-0.04	(-1.05)
Gender			0.31	(1.84)
Age			0.00	(0.46)
Education			-0.07	(-0.42)
Pol. interest			0.03	(0.80)
Income			-0.03	(-0.15)
Constant	-0.19	(-0.97)	-0.59	(-1.49)
One off payment, repayable with low interest after 5 years				
Self-employed	-0.21	(-0.52)	0.12	(0.24)
French	-0.95^{*}	(-4.68)	-0.97^{*}	(-4.35)
Left-right	0.07	(1.89)	0.09^{*}	(2.10)
Gender			0.35	(1.86)
Age			0.01	(1.04)
Education			0.11	(0.58)
Pol. interest			-0.01	(-0.30)
Income			-0.05	(-0.25)
Constant	-0.81^{*}	(-3.57)	-1.34^{*}	(-3.03)
Observations	8988		7530	
Respondents	1498		1255	
Model p-value (chi-sq.)	0.000	(81.18)	0.000	(111.02)

z statistics in parentheses
* $p < 0.05$
